# Annual Address of the President of the Erie County Medical Society, Delivered Jan’y 8th, 1855

**Published:** 1856-02

**Authors:** James P. White


ART. IV.—*Annual Address of the President of the Erie County Medical Society, delivered Jan'y 8th*, 1855. By James P. White, M. D., President.

In accordance with a wholesome rule of this society, I shall claim your indulgence but for a short time before retiring from the position to which, by your partiality, I have been called. Perhaps no greater service could be rendered the profession, than by pointing out the source and the manner in which may be diminished the frequency of quarrels between its members, and the acerbity of feeling thereby engendered.

If in the accomplishment of this most desirable purpose we can improve the pecuniary condition of the profession as a whole, and render its different members more independent of the world, and can unite them in bonds of mutual dependance upon each other, an additional benefit will thereby be conferred. It is humbly hoped that the execution of the project to which your attention is now briefly invited, would in some degree contribute to these important ends.

Preliminary to the discussion of the utility of the scheme to which your attention is invited, let us inquire into the causes of the greater frequency of differences among doctors than among the members of other professions, or among men of business. Have they a larger innate share of original sin than the rest of the human family? If not, is there anything in the study of the sciences upon which medicine is based, or in its practical exercise, calculated to engender hostility to mankind or foster a turbulent spirit? Clearly the reverse. Indeed there is little risk in making the assertion that, ordinarily, so far as his intercourse with all other members of the community, except his fellow laborer, extends, the medical practitioner is an example of amiability, and most free from litigious inclinations. Whence then arises this evil which is truly styled the "opprobrium of our profession?"

Among the negative causes, and one to which our present remarks will be confined, may be mentioned the isolated life (so far as relates to his fellows) which necessarily results from the practice of medicine, and the infrequency of professional reünions for any purpose. No men live more harmoniously than medical students. Brought as they are into intimate relations several times during the day, and without the controlling restraint of home, and at an age when the blood courses with most rapidity, how seldom do serious differences arise! and how brief, ordinarily, are they in duration! The gravest offences among them are forgotten after a few interviews, competitors though they may be called, in scientific acquisitions. Is it possible that conferring the Degree of Doctor in Medicine endows its recipient with belligerent tendencies to which he has throughout life been a stranger? His studies, if rightly pursued, whilst they have been highly attractive in character, affording the amplest range for the exercise of the highest intellectual development, were also calculated to develop the best, the most benevolent feelings of his nature. Their object contemplates the well-being of our fellow creatures, the lessening of human suffering, and the extension of human existence. His sources of thought, and objects for reflection continue the same. Now, however, instead of meeting several times daily, he sees his old fellow student, who has become a neighboring practitioner, but seldom, and and then for a brief period only. True, he hears from him often, but chiefly through distorted media. Envy is excited through exaggerated reports of his success. Slight detractions are indulged by both in the presence of mischievous busybodies, which are repeated with embellishments until each considers himself aggrieved, and a total suspension of intercourse is but too frequently the result. Now these men are possessed with infirmities and passions as other men, and they can also claim to exercise toward the world a charity as catholic and pure as their neighbors in the law, who under similar circumstances would soon be brought into contact in the prosecution of their professional duties, and after a day or two spent in controversy in behalf of their clients, would separate, having forgotten the slanderous report with which busy rumor had perplexed them. Let us, then, avail ourselves of every useful purpose for meeting each other face to face, when each may read mirrored forth the generous purposes which animates his fellow, and forget the prejudice which otherwise would have haunted him through life.

Without permitting any collateral or incidental benefits to engross farther attraction, your serious consideration is asked for the project of establishing in this city a society for the relief of the widows and orphans of medical men. This may at first seem utopian, but dismiss it not hastily. What object can make a stronger appeal to the generous impulses of the profession than the establishment of a society among its members for the relief of those held most dear, and who may at any time, by circumstances beyond control, be left dependant. From whom would relief be so gratefully received as from the fraternal hand of a sympathising fellow? It is analogous to effecting an insurance in a company which pays no salaries to its managing officers, whose funds are watched and invested with a care which is stimulated to vigilance by every motive of honor and fraternal interest, and which invites donations from liberal and wealthy members of the profession, thus adding to the capital stock without levying contributions upon the insured. Besides, by this method of mutual insurance among the members of a noble profession, created for the protection of its own members, a community of interest and sentiment will be secured which would be alike salutary upon those who were engaged in its promotion, upon all who are recipients of its benefits, and in inspiring the respect of all beholders. It has long been the wish of your speaker that a nucleus for the organization of this noble purpose might be commenced, and it is his conviction that the time for action, if it has not already arrived, is certainly not distant.

In many of the larger cities we find them already established, and their usefulness fully demonstrated. In New York a society for this most benevolent purpose, was instituted in 1842, and incorporated in 1843.

The New York society, which is believed to be founded upon principles analogous to those of a similar character in other cities, is composed of three classes or kinds of members: members, life members, and benefactors.

The members consist of physicians and surgeons residing in and near New York, holding regular licenses to practice under the laws of the state of New York, and of the physicians and surgeons of the army and navy of the United States. Every person desiring to become a member of the society, is required to sign a declaration to that effect, setting forth his place of abode, whether single or married, and if he has children giving their number, names and ages. To this declaration there must be annexed a certificate signed by at least two of the members of the society, vouching for the good standing and character of the applicant.

The candidate, thus recommended, must be proposed at one meeting of the board of managers, and balloted for at the next; and in *every notice* to attend such meeting, the names of the persons to be balloted for is expressed. In this way the society could scarcely fail of being protected against the introduction of unworthy men.

Again, all except life members and benefactors, are required, for the purpose of creating a fund, to pay an initiation fee of ten dollars, and also two semi-annual contributions of five dollars each. These sums so paid for twenty years, constitute him a life member, when all farther contributions are optional. The sum of one hundred dollars paid at any time within three months after the date of his election, will also constitute the subscriber a member fór life, and exempt him from future payments.

The payment of $150, by any person, constitutes him a benefactor of the society. And any member of the medical profession thus contributing on being duly proposed and approved of by ballot, is entitled to all the rights and benefits of life membership.

You will perceive that great care is exercised in the introduction of members, and any person omitting to make his payments for a certain length of time, becomes "*ipso facto*" disqualified for membership, forfeiting such sums as he has paid. It however remains discretionary with the board of managers, to restore such person to the privileges of membership upon his making payment of all arrears of dues, and satisfying them of his good faith. It is also provided by the laws of this society, that any quackish or unprofessional conduct shall, after due notice, if persisted in, forfeit membership and all right, title and claim to any portion of the funds, property or benefits of the society. It should, however, be remarked, that removal from the limits of the society, or relinquishing the practice of medicine, does not deprive the member of any of the rights and privileges to which he was entitled.

The affairs of this society are under the direction of a board of managers, consisting of a President, Vice-president, Treasurer, and twenty-one managers. The latter body is divided into three classes of seven each, which in turn serve four months each.

The Treasurer, before entering upon the duties of his office, is required to give a bond with sufficient sureties, to secure the faithful performance of the trust reposed in him

The funds of the society, now amounting to more than $16,000, are invested in stocks of the United States, of the state and city of New York, and in bonds and mortgages on real estate in the city of New York, and in *no other mode.* It is intended that all sums derived from benefactors and life members, shall go to increase the capital stock, whilst the interest and so much of the annual assessment of members as may be necessary, are applied to the relief of those entitled to its benefits.

The proper subjects of relief are the widows and children of members of the society, who have no estate or income exceeding the value of $200 per annum. These, and under certain circumstances, other members of the family of the deceased are eligible to receive assistance as the board of managers shall believe the state of the funds will permit.

Although the general intention of the society is to relieve the widows and children of medical men, it is nevertheless lawful for the board of managers to allow temporary relief, if they deem it necessary, in case any member becomes incapacitated from attending to his business.

At the anniversary meeting, in addition to the election of officers and the transaction of other business, a dinner by the members, the expenses of which are paid by the sale of tickets, concludes the day, and contributes to the good feeling existing among its members. This society numbered among its benefactors and life members, two years since, more than sixty of the best men in that metropolis. Here are enrolled the uames of Mott, and Stevens, and Parker, and Wood, and Smith, and Watson, and Griscom, and Delafield and others like them.

Among a much longer list of members may be found the names of such men as J. Kearney Rodgers, and Brown, and Washington, and Stearns, and Campbell, and many others who have, since their union with this society, dissolved connection with all temporal associations, and left all that was most dear in life to the care and sympathy of their fellows.

This, gentlemen, is the kind of society for which your attention is claimed, and in the establishment of which you are urged actively to participate. What can be more noble or humane? Some may think we are not yet sufficiently numerous in this city to undertake an organization for this purpose. This may be true. For my own part, however, I believe that taking the initiative at this time would not be premature. The city is rapidly increasing, and with it the members of the profession in a still greater ratio. Make the beginning, sow the seed, and in time it will bring forth mature and abundant fruit. The organization once accomplished, will claim a share in all the bequests of the liberal and philanthropic. It will especially commend itself to the generosity of medical men and receive largely of their bounty.

Finally, gentlemen, I leave the subject in your hands, claiming for it such consideration on your part as its merits alone may seem to you to demand.

Craving your forbearance for the many defects of the preceding remarks, hastily thrown together, and thanking you for the manifestation of confidence and respect which by your suffrages you have exhibited in my election to the presidency, and thanking you also for the uniform courtesy extended to me whilst engaged in the discharge of official duties, and bespeaking your lenient judgment for the imperfections which I am conscious have attended my best efforts to serve you, I shall be happy to transmit the insignia of office to a more able successor.

